# Pediatric scurvy case report: a novel presentation with deep vein thrombosis secondary to large bilateral spontaneous iliac subperiosteal hematomas

**DOI:** 10.1186/s12887-024-04579-4

**Published:** 2024-02-16

**Authors:** Henry Charles de Boer, Jasdev Singh Sawhney

**Affiliations:** Doncaster and Bassetlaw Teaching Hospitals NHS Foundation Trust, Doncaster, UK

**Keywords:** Vitamin C deficiency, Scurvy, Autism, Deep vein thrombosis, Subperiosteal hematoma, case report

## Abstract

**Background:**

Scurvy is an uncommon disease in developed countries caused by deficiency of vitamin C. We present a case of scurvy in a 14-year-old male with autism with both novel presentation and imaging findings. This case had the novel presentation of lower limb deep vein thrombosis (DVT) secondary to compression of the external iliac vein from large bilateral iliac wing subperiosteal hematomas. Subperiosteal hematoma is a well-recognised feature of scurvy but large and bilateral pelvic subperiosteal hematoma causing DVT has not previously been described.

**Case presentation:**

A 14 year old Caucasian male with background of autism and severe dietary restriction presented with lower limb swelling and immobility. He was diagnosed with lower limb DVT. Further investigation revealed an iron deficiency anaemia, and he was found on MRI to have large bilateral subperiosteal iliac hematomata causing compression of the iliac vessels. He improved following treatment with vitamin C replacement and follow-up imaging demonstrated resolution of the DVT and hematoma.

**Conclusion:**

DVT is rare in children and when diagnosed should prompt investigation as to the underlying cause. This case demonstrates an unusual cause of DVT and as an unusual presentation of paediatric scurvy.

## Background

Scurvy is a disease caused by deficiency of vitamin C. Scurvy is uncommon in developed countries and the clinical manifestations are non-specific and wide ranging, including bleeding, musculoskeletal pain, iron deficiency anaemia and rash. Therefore, vitamin C deficiency is frequently initially misdiagnosed or the diagnosis delayed. We present a case of scurvy in a teenage boy with a background of autism, who presented with a two month history of immobility and acute lower limb swelling due to a deep vein thrombosis. MRI of the pelvis and hips revealed large bilateral subperiosteal iliac hematomas. Further interrogation of the clinical history and examination findings, together with subsequent improvement following vitamin C replacement are consistent with the diagnosis of scurvy.

To the authors knowledge, this is the first reported case of large spontaneous bilateral iliac bone subperiosteal hematomas in scurvy and the first case of deep vein thrombosis caused by pelvic subperiosteal hematoma. Our objective is to highlight this case to musculoskeletal radiologists to improve the awareness of scurvy as a cause of spontaneous bleeding and particularly to emphasize the importance of early clinical suspicion in the context of children or adults with developmental issues, cognitive impairment, or severe dietary restriction for any other reasons.

## Case presentation

A 14-year-old boy with a diagnosis of autism and pathological demand avoidance with severe dietary restriction, presented with their parents to the local Children’s Assessment Unit with acute swelling of the left foot following a two month history of progressive immobility. The patient denied specific pain in the left lower limb but did complain of generalised muscle aching.

The progressive immobility was first thought to be psychosomatic, having had a similar episode two years prior trigged by illness of a pet and resolved following physical therapy. The patient had been seen by physiotherapy and orthopaedic teams prior to this admission.

**Fig. 1:**
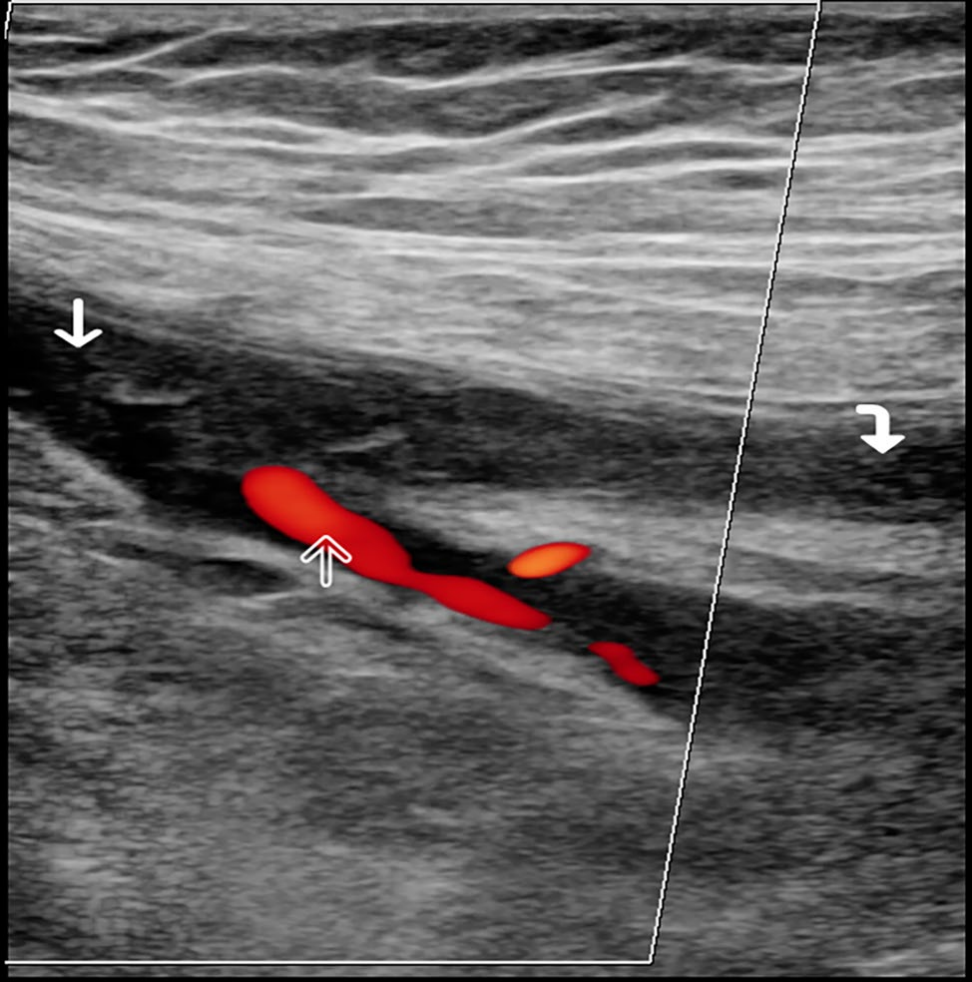
Colour doppler ultrasound of the left common femoral vein bifurcation (solid white arrow proximal). Echogenic material is seen occluding the lumen of the vessel with absence of colour flow in keeping with occlusive deep vein thrombosis which extends into the superficial femoral vein (curved arrow). There is colour doppler flow seen at the origin of the profunda femoris vein (open arrow) indicating partial patency although non-occlusive echogenic thrombus is seen here

**Fig. 2:**
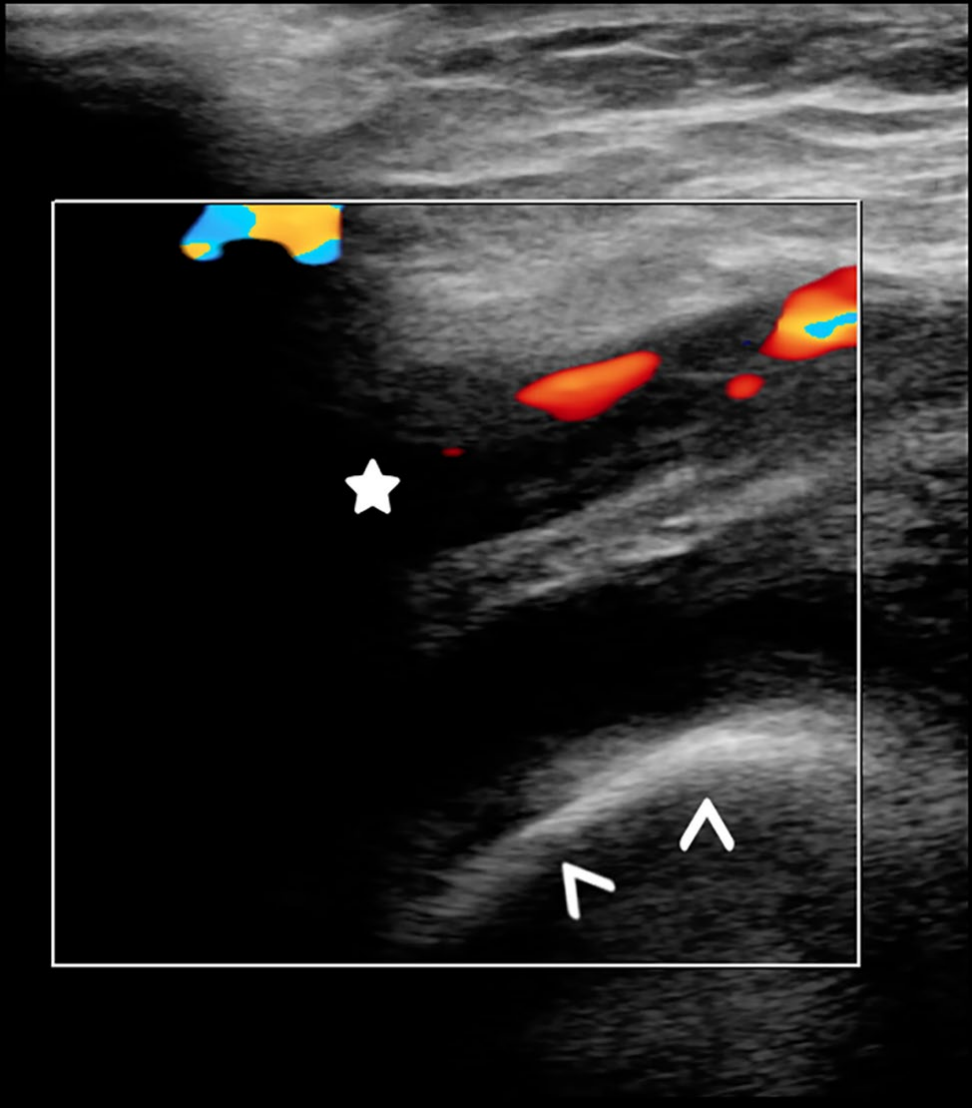
Colour doppler ultrasound of the left common femoral vein (star marks proximal) demonstrates extension of the thrombus proximally from the level of the femoral head (arrow heads). The full proximal extent is not seen on these images

Doppler ultrasound examination of the lower limbs demonstrated extensive deep vein thrombosis in the left superficial femoral vein extending into the left common femoral vein, the proximal extent was not seen [Fig. 1, Fig. 2]. More proximal assessment of the distal external iliac veins was not possible because the patient declined examination of this intimate region. Treatment with low molecular weight heparin, subcutaneous injection of enoxaparin 1 mg/kg twice daily was commenced and then titrated based on anti-Factor Xa activity. Ultrasound examination of the abdominal organs was normal. There was no apparent cause for the deep vein thrombosis, although it was considered that the immobility could be a contributing factor.

**Fig. 3:**
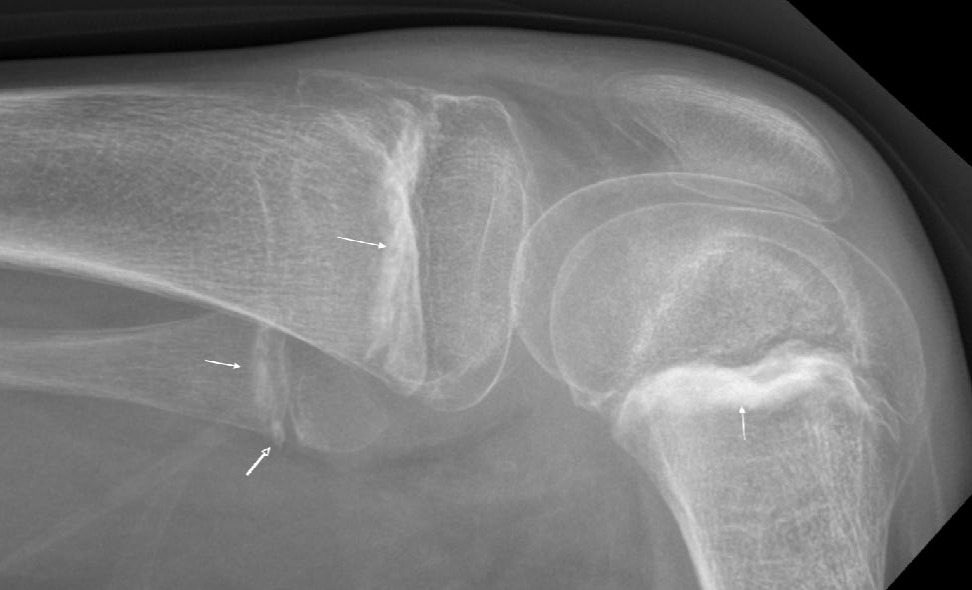
Anteroposterior radiograph of the left wrist. Although initially interpreted as normal demonstrates classical radiographic features seen in scurvy: dense metaphyseal bands known as Frankel lines (solid white arrow) and metaphyseal spurs known as Pelkin spurs (open white arrow). Symmetrical appearances were seen in the contralateral wrist

**Fig. 4:**
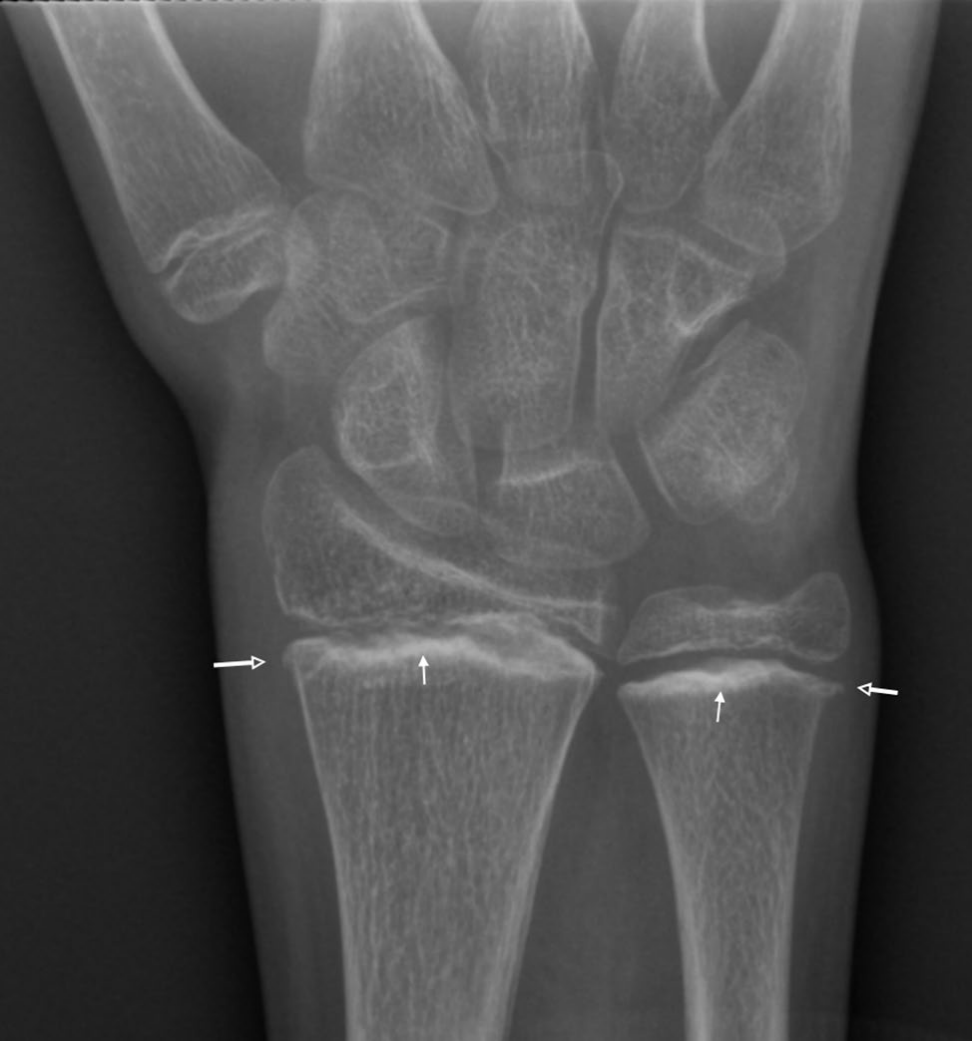
Horizontal beam lateral radiograph of the right knee. Although initially interpreted as normal demonstrates classical radiographic features seen in scurvy: dense metaphyseal bands known as Frankel lines (solid white arrow), also with underlying band of lucency known as Trümmerfeld zones, and metaphyseal spurs known as Pelkin spurs (open white arrow)

Physical examination was difficult so further investigations were requested. Radiographs of the upper and lower limbs were initially interpreted as normal, the metaphyseal findings typical of scurvey were recognised during a second review for the pediatric multidisciplinary meeting [Fig. 3, Fig. 4]. Laboratory testing demonstrated a severe anaemia with microcytosis and low ferritin in keeping with a severe iron deficiency anaemia. The patient also had folate deficiency, a mildly increased erythrocyte sedimentation rate and c-reactive protein but normal white cell counts. A serum autoantibody and rheumatology screen was negative [Table 1].

**Table 1:** Laboratory investigations performed on admission. Results outside of the normal range are indicated in bold type and underlined

	Reference values	Result
**Full blood count**		
Hemoglobin, g/dL	12.6–18.0	**4.8**
White cell count, x10^9/L	4–12	5.9
Haematocrit	0.42–0.52	**0.18**
Mean cell volume, fL	78–100	**73**
Mean corpuscular hemoglobin, pg	27–32	**20**
Mean corpuscular hemoglobin concentration, g/L	310–350	**274**
Platlets, x10^9L	140–450	368
**Electrolytes and Urea**		
Creatinine, mg/dL	0.45–0.94	**0.36**
Blood urea nitrogen, mg/dL	10.0–20.0	10.1
Sodium, mmol/L	133–146	**128**
Potassium, mmol/L	3.5–5.3	3.5
**Bone profile and liver function**		
Total protein, g/dL	6.0–8.0	**5.2**
Albumin, g/dL	3.0–5.0	**2.6**
Globulin, g/dL	2.3-4.0	2.6
Alk. Phos, IU/L	60–425	111
Adjusted Calcium, mmol/L	2.29–2.63	2.31
Phosphate, mmol/L	0.9–1.8	1.3
ALT, U/L	0–55	6
Total bilirubin, umol/L	0–21	13
**Inflammatory markers**		
C-reactive protein, mg/L	0–5	**14**
Erythrocyte sedimentation rate, mm	1–10	**44**
**Clotting Tests**		
Prothrombin Time, s	10-14.1	**15.9**
APTT, s	24.6–38.4	25.3
Fibrinogen, g/L	1.7–5.3	2.1
D-Dimer, ug/mL	0.0-0.5	**4.92**
**Iron profile**		
Iron, ug/dL	64.8-174.9	**22.4**
Transferrin, mg/dL	200–320	**160**
Transferrin sat, %	15–45	24
25-OH Vitamin D, ng/mL	> 20.0	55.7
Thyroid stimulating hormone, mU/L	0.47–3.41	1.13
Folic acid	3.1–20.5	< 1.6
Vitamin D12, ng/L		1267
**Rheumatology**		
Anti-CCP, U/ml	0–10	< 1
Rheumatoid Factor, IU/mL	0–30	20
Autoantibody screen		NEGATIVE
**Urine culture**		No growth

**Fig. 5:**
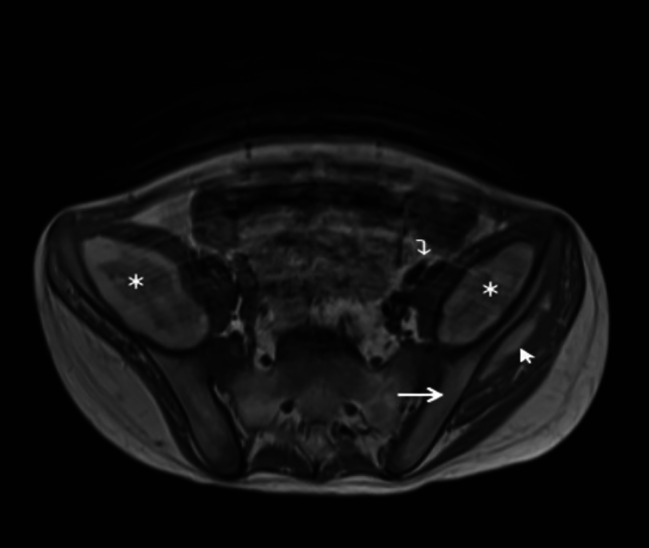
MRI of the pelvis, axial T1. Bilateral high signal ovoid collections along the medial margin of the iliac wing (long arrow) bilaterally represent subperiosteal hematoma (marked with *). Displacement of the iliac vessels (curved arrow) and iliopsoas muscles. Smaller high signal collection seen along the lateral margin of the left iliac wing deep to the gluteal muscles (short arrow)

**Fig. 6:**
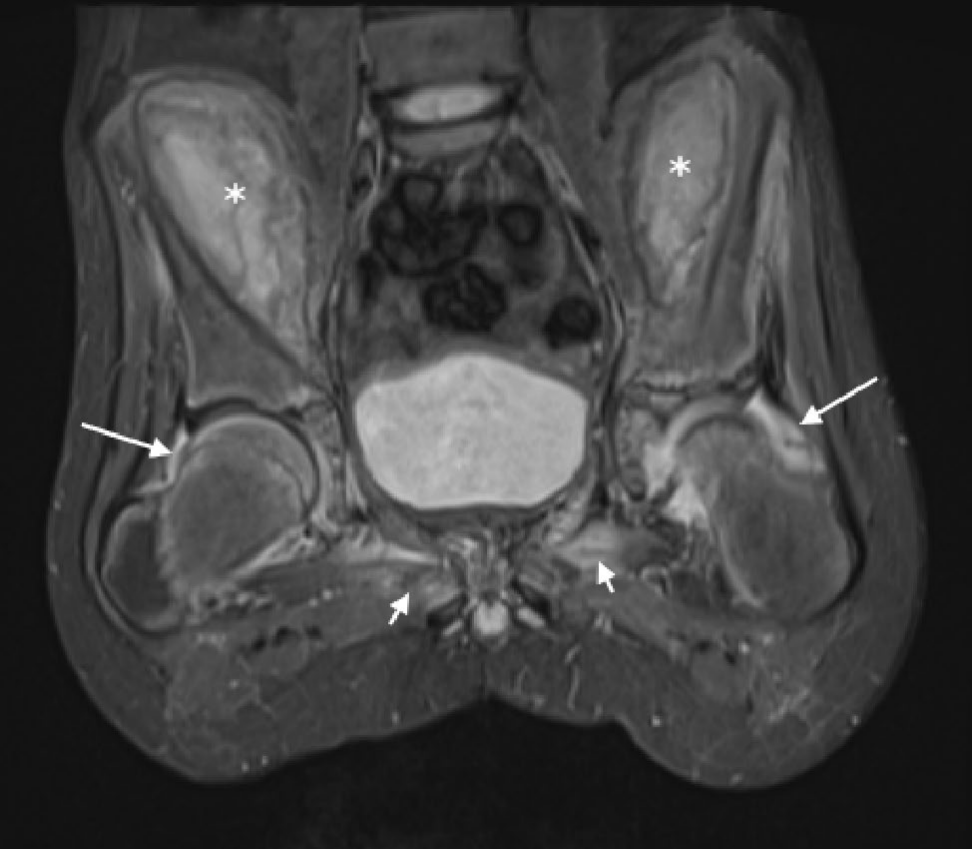
MRI of the pelvis, coronal T2 fat saturated. Mixed but predominantly high signal collections are seen along the medial margin of the iliac wings bilaterally (marked with *). Small bilateral hip effusions (long arrows) and high signal in the adductor muscles (short arrows), more so on the left

MRI of the head, spine, pelvis and hip was performed. The brain and spinal cord were normal. In the pelvis there were large collections, lentiform in shape, along the medial margin of the iliac wings bilaterally. The collections showed mixed high and intermediate T1 and high STIR signal. They measured approximately 9 × 6 × 4 cm (anteroposterior x craniocaudal x width) and displaced the iliacus and psoas muscles medially to compress the external iliac vessels. The signal characteristics and location were in keeping with subperiosteal hematomata. A similar smaller subperiosteal hematoma was seen on the lateral aspect of the left iliac wing, deep to the gluteal muscles. Small bilateral hip joint effusions and high signal in the adductor muscles was also seen. There was no significant bone marrow oedema. [Fig. 5, Fig. 6] Following discussion in the pediatric multidisplinary meeting, scurvy was suggested as the unifying diagnosis after review of the clinical history, blood results, radiograph and MRI findings. It was at this second review that the radiographic features of scurvy were recognised. A reducing regime of oral vitamin C replacement was commenced, starting at 1000 mg once daily, reducing to 500 mg, 200 mg, 100 mg and stopping at 2 weekly intervals.

In the two months prior to this presentation, the patient’s parents reported a significant reduction in the variety of his already narrow diet, mostly eating cheese sandwiches and crackers. Prior to this he was eating jam and toast which would have been a source of vitamin C. His admission weight was 36.45 kg, significantly lower than the 45 kg weight recorded at an outpatient clinic visit two months earlier. During this period, the patient had been complaining of ‘pain all over’ and his mobility had steadily declined from walking to being wheelchair bound. Following subsequent scrutiny of the clinical history, the patient and his family did remember a rash on the lower legs around the hair follicles and some bleeding when brushing his teeth. These features had all resolved by the time of the MRI, probably because the patient had been prescribed a multivitamin on admission, Forceval 1 capsule once daily one hour after food, whichcontains vitamin C.

Serum vitamin C levels prior to vitamin C replacement had not been measured but considering the clinical history, imaging findings and improvement of symptoms following commencement of vitamin C replacement, scurvy is the presumed underlying cause of the spontaneous hematomata. This would also explain the iron deficiency anaemia, both follow blood loss and as a direct effect of vitamin C deficiency on iron metabolism. Compression of the left external iliac vein due to the hematomas was the reason for development of DVT in this child.

Severe anaemia was treated with blood transfusion, 3 units of packed red cells, seeing improvement in hemoglobin from 4.8 to 9.2 g/dL. High dose vitamin C replacement, Forceval one capsule once daily one hour after food, was commenced along with intensive physiotherapy and dietician input. The patient’s mobility improved during his two-week inpatient stay but he did remain dependent on his wheelchair, struggling to extend his legs fully. His physical health and mood also improved during this time. Enoxaparin was changed to oral rivaroxaban, 15 mg once daily, on discharge.

Follow-up doppler ultrasound of the lower limbs demonstrated resolution of the DVT at 12 weeks, a repeat haemoglobin was normal at 12.7 g/dL and the patient’s ESR and CRP had normalised. Serum vitamin C was normal at this time.

**Fig. 7:**
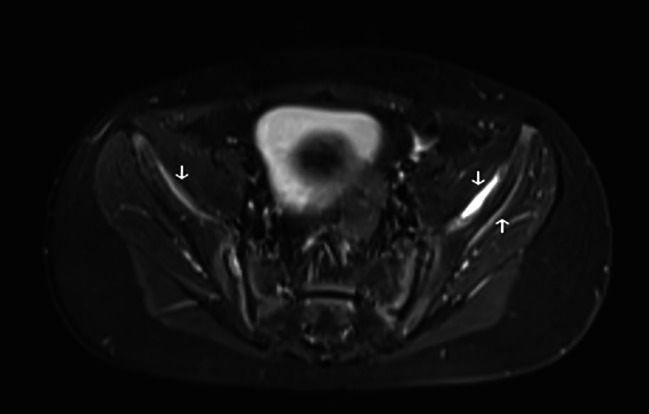
Follow-up MRI at 5 months post presentation, axial T2 fat saturated. The previously seen iliac subperiosteal hematoma have significantly reduced in size, there are thin residual high signal collections (white arrows)

Follow-up MRI scan 4 months following the initial presentation demonstrated a significant reduction in subperiosteal iliac hematomata with only thin residual collections along the medial and lateral iliac margins. [Fig. 7] The patient continues to take the multivitamin daily and will do so indefinitely to prevent future vitamin deficiencies. Rivaroxaban was discontinued at 4 months post initiation. The patient’s mobility has continued to improve, and he is now mobilising short distances.

## Discussion and conclusions

Vitamin C is an essential nutrient, an important cofactor for the biosynthesis of collagen in blood vessels, skin amongst other tissues. Vitamin C also promotes iron absorption and transport. Humans cannot synthesis or store vitamin C which means we are reliant of minimum daily dietary intake to prevent deficiency [1]. 

Vitamin C deficiency (< 11µmol/L) is more common in developed countries than most clinicians would think, with a prevalence of 1–16% [2] Severe deficiency is however much less common, its prevalence < 0.01% and typically seen in patients with severe dietary restriction such as children with autism and older adults with cognitive impairment.

Severe vitamin C deficiency can lead to potentially life-threatening haemodynamic instability but has a diverse clinical presentation, usually with multisystem involvement. The most common clinical findings are bleeding tendencies including a petechial rash, perifollicular hyperkeratosis, iron deficiency anaemia, lower limb pain and reluctance to walk.

In this case, the patient had recognised risk factors for vitamin C deficiency, namely autism and a severely restricted diet. Recognition of these risk factors led to the suspicion of nutritional deficiencies as a cause for his symptoms and appropriate early initiation of multivitamin replacement. Non-specific lower limb pain, reduced mobility, petechial rash as present in this case are all well recognised in vitamin C deficiency. Vitamin C deficiency often co-existes with other nutritional deficiencies such as folic acid, vitamin D and vitamin B12. Whilst spontaneous haemorrhage is well recognised manifestation, this is mostly commonly: intra-orbital, subdural or when subperiosteal, in the lower limbs [3–5]. This is, to the authors knowledge, the first reported case of intra-pelvic subperiosteal hematoma due to scurvy.

The MRI findings in scurvy are often non-specific with patchy bone marrow oedema. Finding of subperiosteal hematoma is less common but more specific [6]. The most important consideration of imaging is not in the diagnosis of scurvy but the exclusion of other acute pathologies which could mimic the presentation including haematological malignancy, septic arthritis, insufficiency fracture and slipped upper femoral epiphysis.

Classically described radiograph findings in scurvy include cortical thinning, dense zone of provisional calcification, epiphyseal increased density in a ring around the periphery and transverse lucency in the metaphysis on the diaphyseal side of the dense provisional calcification. In practice these can be difficult to pick up due to the rarity of the condition and are often only appreciated in retrospect after the diagnosis has already been made [7]. 

DVT in children is rare in children, usually caused by the use of intravenous catheters or prothrombotic conditions [8]. Unlike in adults, immobility is not a strongly correlated risk factor. Therefore, DVT in a child with proximal into the iliac vessels should prompt a thorough search to exclude a compressive lesion such as May-Thurner syndrome [9] or as reported by Culler at al, faecal impaction causing gross rectal distention compressing the left external iliac vein in a child [10, 11]. Cases of large periosteal hematoma have been reported, commonly in the long bones [12] ,but this is the first reported case of large iliac subperiosteal hematoma and is a previously unreported cause of external iliac vessel compression and DVT. This casedoes demonstrate the need for cross-sectional imaging of the pelvis in cases were DVT remains unexplained after ultrasound in the absence of haematological conditions.

## Data Availability

The datasets used and/or analysed during the current study available from the corresponding author on reasonable request.
